# Alien or Native? How to Distinguish Feces of Fallow and Roe Deer Using Central Poland as a Case Study

**DOI:** 10.3390/ani12030290

**Published:** 2022-01-25

**Authors:** Jakub Gryz, Dagny Krauze-Gryz, Daniel Klich

**Affiliations:** 1 Department of Forest Ecology, Forest Research Institute, Braci Leśnej 3, Sękocin Stary, 05-090 Raszyn, Poland; j.gryz@ibles.waw.pl; 2 Department of Forest Zoology and Wildlife Management, Institute of Forest Sciences, Warsaw University of Life Sciences WULS-SGGW, Nowoursynowska 159, 02-776 Warsaw, Poland; 3 Department of Animal Genetics and Conservation, Institute of Animal Sciences, Warsaw University of Life Sciences WULS-SGGW, Ciszewskiego 8, 02-786 Warsaw, Poland; daniel_klich@sggw.edu.pl

**Keywords:** pellet group counts, alien species, cervids, feces characteristics, feces morphometry

## Abstract

**Simple Summary:**

Fallow and roe deer are both game species, and therefore it is crucial to monitor their population locally and on a country scale. The method of pellet group count is commonly used for estimating population trends of ungulates; however, in the case of the two species, the misidentification rate can be high due to their similar body size. Our aim was to determine a metrical threshold between pellet groups of roe deer (native species) and fallow deer (alien species) to be applied during fieldwork. We measured the number of feces in the group, the length and width of five randomly selected feces from each pellet group and the length/width ratio. Roe deer pellets were shorter, narrower and less elongated than those of fallow deer; yet, length was found to be the best discriminant. The most accurate threshold was 1.2 cm. The mean number of pellets in a group was lower for roe deer than for fallow deer. A value of 50–52 pellets best differentiated between the two species. We therefore concluded that, on the basis of morphometric measurement, it is possible to distinguish roe and fallow deer feces.

**Abstract:**

The method of pellet group count is commonly used for estimating population trends of ungulates; however, in the case of species of similar body size, the misidentification rate can be high. Our aim was to find a metrical threshold between pellet groups of roe deer (native species) and fallow deer (alien species) to be applied during fieldwork. The study was conducted in spring 2020 and 2021 in central Poland (lowlands) in areas were only roe or fallow deer occurred. We measured the number of feces in the group, the length and width of five randomly selected feces from each pellet group and the length/width ratio. Roe deer pellets were shorter, narrower and less elongated than those of fallow deer; yet, length was found to be the best discriminant. The most accurate threshold was 1.2 cm, i.e., 12–15% of pellets were over/below this value. The mean number of pellets in a group was lower for roe deer (39.6, SE = 1.6) than for fallow deer (64.5, SE = 1.5). A value of 50–52 pellets best differentiated between the two species. To conclude, combining these two measurements could be an objective method to distinguish between pellet groups of the two species.

## 1. Introduction

The method of pellet group count is commonly used by hunters, wildlife managers and scientists worldwide for estimating the density, population trends, age structure and habitat selection of ungulates [1,2,3,4,5,6,7,8,9,10]. This method is cheap and does not require any special equipment. Nevertheless, distinguishing pellets groups of different species might be difficult during fieldwork, especially when similar species coexist, i.e., misidentification rates are highest between ungulates that are from the same family and of similar body size [11], which is the case of roe deer (*Capreolus capreolus*) and fallow deer (*Dama dama*). Popular guidelines, e.g., Refs. [12,13,14,15] did not offer clear practical answers for how to determine the feces of roe and fallow deer. At the same time, a recent genetic study showed that errors in pellet field identification might be significant [11].

Fallow deer is an alien species in the fauna of Poland and most of Europe. Its natural range covers Asia Minor, the Middle East and Macedonia [16,17,18]. In the Middle Ages, fallow deer became a popular species in Europe, and, in the following centuries, the abundance and territorial range increased, and now this deer occurs on all continents, except for Antarctica. It is a popular game, livestock and ornamental animal [18,19,20,21,22] and is numerous in Poland [23,24,25]. 

In past decades, the abundance of fallow deer has increased in Poland due to intentional introductions by game managers, accidental introductions of animals escaping from captivity and transboundary immigration from the Czech Republic and Germany. The abundance of free-living fallow deer is impossible to estimate precisely but is likely somewhere between 30,000 to 70,000 individuals in the spring [26]. However, by comparing available data, a clear population trend is apparent. 

Poland is divided into 4965 hunting districts. In 2001, fallow deer occurred in 6% of those, while in 2020, they occurred in 19%. Similarly, in the national parks: in 2006, the species was reported from four parks, while in 2021 from seven parks. Fallow deer are also found in cities and other areas not included in hunting districts and national parks [27]. In the twentieth century, the range of the species encompassed mostly western Poland. Currently fallow deer are present in all voivodeships [24,25,26,28]. The hunting of fallow deer has grown substantially, i.e., in 1990, 1700 individuals were harvested, while in 2018, this number was more than 10,000 [26,28]. 

Roe deer, in turn, is the most abundant angulate in Poland as reported from the whole country [29] occurring in all hunting districts, national parks and also living (and increasing its abundance) in urban green spaces [30,31]. Its abundance in the whole country is estimated to exceed over 900,000 individuals [26] and is stable/slightly increasing [32]. The two species co-occur in many regions, as they are sympatric and inhabit the same habitats.

For decades, the influence of fallow deer on native fauna and flora has been dis-cussed, with no clear conclusions thus far [25,33]. One of the very few studies conducted in Poland showed a food niche overlap (exceeding 50%) between fallow deer and native cervids, which pointed to important competition between the species [34]. 

According to other papers published in a popular hunting magazine only with a very high density of fallow deer, can the species negatively affect native fauna [35,36,37]. Nevertheless, as shown by an Italian study, roe deer avoided areas intensively penetrated by fallow deer, and, with a high density of alien species, the roe deer density decreased [38]. Other studies performed outside of Europe showed that introduced fallow deer negatively influenced the native fauna and flora [21,22,39,40].

As both fallow and roe deer are game species, it is crucial to monitor their population locally and on a country scale. It is important to also monitor if and how alien species affect the abundance of native species (i.e., roe deer). Therefore, the aim of our study was to find a metrical threshold between pellet groups of roe deer and fallow deer on the basis of feces measurements that may be applied in the field.

## 2. Materials and Methods

The field work was done in central Poland (the lowlands) in areas where either roe or fallow deer occurred (i.e., only one species was present in a sampling site), with no other deer species. Roe deer feces were measured in the Arboretum of Warsaw University of Live Sciences near Rogów village (arboretum.sggw.pl, accessed on 10 September 2021). This area was mostly fenced; however, a few individuals of roe deer (♂, ♀ and two or three unidentified individuals) existed there. 

Animals occupied mainly unused part of the arboretum, which constituted 15 ha of mature pine (*Pinus sylvestris*) wood, with an admixture of oaks (*Quercus* spp.), hornbeam (*Carpinus betulus*) and firs (*Abies* spp.). There was no supplementary feeding. Additionally, we measured feces in a small (130 ha) forest complex located nearby (i.e., less than 1 km). The forest was surrounded by arable lands and located to a distance to other wooded areas. There, the density of roe deer was relatively high, reaching 60–70 ind./100 ha in the spring [41]. 

Stands consisted mainly of pines and oaks. According to our knowledge (based on long-time fieldwork conducted in this area) and information delivered by a local game manager [42], fallow or red deer were not present in this forest complex. Supplementary food (i.e., hay) was delivered only in winter. Fallow deer feces were measured in an ex situ breeding site that was located in Dobieszyn Forest District. Fallow deer were bred there to be introduced to the surrounding hunting grounds. 

During our study, there were 43 individuals (six ♂ and ♀ with calves), living in a 60 ha fenced forest area, with a large meadow in the center of the enclosure. In order to provide water for animals, an artificial pond was placed within the enclosure. Stands consisted mainly of pines and oaks. During the winter, hay was delivered and (rarely) other food types. No other deer species were present in the enclosure.

Feces measurements were conducted in the spring (March–early April) of 2020 and 2021. In central Poland, feces accumulate during the winter season (November–March) and decomposition starts in late April [1]. We measured the number of feces in the group and the length and width of five randomly selected feces from each pellet group (100 pellet groups of one species in total). 

As some pellets were soft and became distorted when measured with the aid of a caliper, we put a pellet on a steel tape measure and recorded its measurements with the precision of 1 mm. To increase the likelihood of sampling different individuals, we placed at least 50–100 m between measured samples. To measure the pellet shape, we calculated the length/width ratio.

Due to the need for raw measurements to be used in the field assessment of the species, differences in the length, width and length/width ratio calculated for single pellets were compared with the Kruskal–Wallis test (the data did not follow a normal distribution—Shapiro–Wilk W test, see Supplementary Materials). The number of pellets in a group was compared with the Kruskal–Wallis test. The mean values (with 95% CI) are given.

To verify relations between pellet measurements, we performed three generalized linear mixed models with pellet group ID as a random effect to account for repeated sampling within groups. In Model 1, the dependent variable was the feces length, and the independent variables were the species (SPECIES), feces width (WIDTH), number of pellets in a group (nPELLETS) and interaction of width and species (WIDTH*SPECIES). 

In Model 2, the dependent variable was feces width, and the independent variables were the species (SPECIES), feces length (LENGTH), number of pellets in a group (nPELLETS) and interaction of length and species (LENGTH*SPECIES). In Model 3, the dependent variable was the number of pellets in a group, and the independent variables were the species (SPECIES), feces length (LENGTH) and feces width (WIDTH). We used a gamma distribution with a log link function in all models. In all four models, we performed a model selection based on our hypothesis [43]. All possible model permutations were performed and, finally, the models were ranked according to their AICc values. The principle of model selection was ΔAICc > 2.

To verify the possibility of a clear separation of species based on raw measurements, we used mixed effects logistic regression (Model 4 (RAW_A_)), where the dependent variable was the species. In this model, the roe deer was marked as 0, and the fallow deer was marked as 1. The explanatory variables were the analyzed measurements: LENGTH, nPELLETS and WIDTH. The IDs of groups were set as a random effect to account for repeated sampling in groups. We verified variables with regard to the *p*-value and standard error. 

Then, we built Model 5 (RAW_B_) with two variables, which best explained the separation of the species and were also the two least overlapping measurements: LENGTH and nPELLETS. A dependent variable and random effect was similar to the Model 4. Due to the fact that the AICc of the models was higher than of the null model, the similar models were performed without the random effect. To check whether averaged values could better differentiate species, we used logistic regression (Model 6 (MEAN_A_)), in which the dependent variable was the same as in Model 6. However, the feces length (mLENGTH) was calculated for the mean of five pellets selected from a group, while the nPELLETS in the group was the same value as in Model 4 and 5. We compared the regression with the number of correctly classified cases, AUC and odds ratios for explanatory variables. Finally, we built two logistic regressions, separately for length of the feces and number of pellets (Model 7 (MEAN_B_) and Model 8 (MEAN_C_) with only mLENGTH or nPELLETS as an explanatory variable. 

To confirm the logistic regression results, we used discriminant analysis with “leave-one-out” (jackknifed) cross-validation on the morphometric variables (i.e., the mean length and width calculated for five pellets selected from a group and number of pellets in a group) to assign pellet groups to species. The Kruskal–Wallis test and discriminant analysis were performed in Past 4.05 [44] software, and generalized linear mixed models and logistic regression models were performed in SPSS statistics 26.0 (IBM, Armonk, NY, USA).

## 3. Results

**Figure 1:**
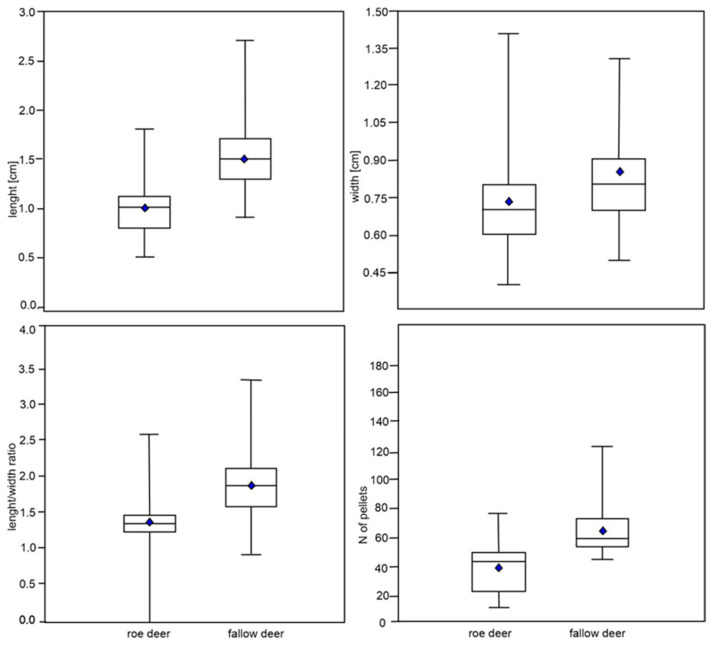
Comparison of morphometric measurements on 100 pellet groups (five pellets were randomly selected from each pellet group) of roe deer and fallow deer: pellet length, pellet width, pellet shape (length/width ratio) and the number of pellets in a group. The median is shown with a horizontal line inside the box, and the 25–75 percent quartiles are drawn using a box. The minimal and maximal values are shown with short horizontal lines. The mean values are shown by blue diamonds.

Roe deer pellets were significantly shorter (1.0 cm, CI = 0.96–1.00) than those of fallow deer (1.5 cm, CI = 1.50–1.54), (Kruskal–Wallis test, H = 547.5, *p* < 0.001). The difference in the width of roe deer and fallow deer pellets was very small (i.e., approx. 1 mm) but still statistically significant (H = 102.6, *p* < 0.001). Fallow deer pellets were more elongated in shape, i.e., the length/width ratio was higher than the ratio calculated for roe deer (H = 453.8, *p* < 0.001). The mean number of pellets in a group was lower for the roe deer and equaled 39.6 (median = 43.5, min = 12, max = 76, CI = 36–43), compared with for the fallow deer (64.5, median = 59, min = 45, max = 123, CI = 61–67) (H = 93.1, *p* < 0.0001) (Figure 1).

All measurements showed statistically significant differences between species. In the case of the pellet length and width, the interaction was also statistically significant, indicating that, in the case of roe deer, the increase of the pellet width with its length was more pronounced. Interestingly, nPELLETS was rejected in the model selection process in Model 1, Model 2 and Model 3. Moreover, in Model 3, selection excluded the feces length and width (Table 1, Supplementary Material Figure S2).

**Figure 2:**
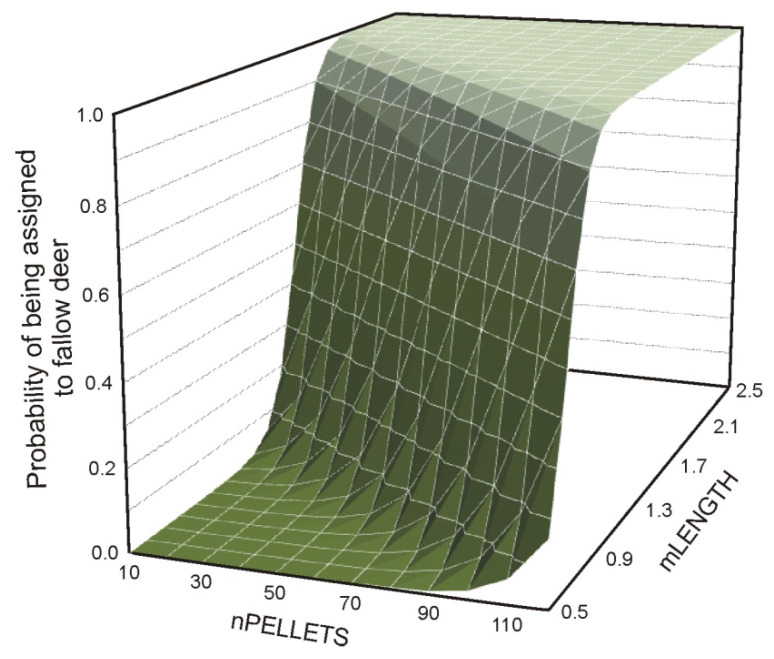
Probability of assignment of feces to fallow deer based on the mean length of a pellet (mLENGTH) and the number of pellets in a group (nPELLETS) in logistic regression (Model 6 (MEAN_A_)).

The mixed logistic regression model, including all variables (Model 4 (RAW_A_)), indicated that LENGTH and nPELLETS differentiated between the species better than WIDTH. This was shown by the *p*-value and standard error of the B coefficient (Table 2). Both regression Models 5 and 6, with LENGTH (or mLENGTH) and nPELLETS, were based on raw data, and the mean pellet length significantly separated the species. Both explanatory variables were significant. Nevertheless, the Model 6 (MEAN_A_), which was based on the mean values (calculated for five pellets in a group), presented a much higher odds ratio for the pellet length when compared to Model 5 (RAW_B_). A more rapid increase of the probability of pellet assignment to fallow deer was seen in the case of the model based on mean values rather than on raw values (Table 2). Moreover, Model 6 presented a higher number of correctly classified cases than Model 5 (93% and 91.1%, respectively), and the model that was based on mean values (Model 6) showed higher AUC values then model based on raw data (Model 5) (0.99 and 0.97, respectively). In Model 6 (MEAN_A_), the increase of the probability of feces assignment to the fallow deer was more pronounced for mLENGTH than for nPELLETS (Figure 2). 

**Figure 3:**
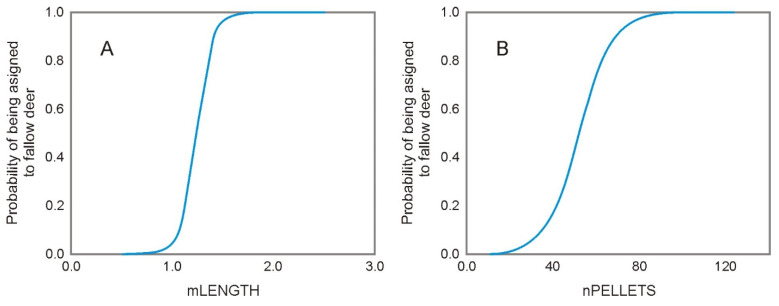
The probability of assignment of feces to fallow deer as based on (**A**) the mean length (mLENGTH) of a pellet or (**B**) the number of pellets (nPELLETS) in a group in logistic regressions (Model 7 (MEAN_B_) and Model 8 (MEAN_C_).

When using only length as an explanatory variable (Model 7 (MEAN_B_)), the odds ratio was only slightly lower (Table 2), the number of correctly classified cases decreased to 89.1%, and the AUC was similar to Model 6 (0.97 vs. 0.99). When taking into account only pellet length (mLENGTH and, regardless, nPELLETS) the probability of assignment of feces to fallow deer drastically increased towards 1 after reaching a value of 1.2 cm (Figure 3A). 

Model 8 (MEAN_C_), which was based solely on the number of pellets in a group (nPELLETS), presented a lower percent of correctly classified cases (82.1%) and lower AUC value (0.893) comparing to Model 7 (MEAN_B_), with mLENGTH as an explanatory variable. When only the number of pellets was taken into account (nPELLETS), the probability of assignment of feces to fallow deer increased towards 1 less drastically than in the case of the pellet length. For 52 pellets in a group, the probability of feces being assigned to fallow deer exceeded 0.5 (Figure 3B).

Discriminant analysis, based on the morphometric data, assigned the correct species to 94.03% (i.e., 93% of roe deer samples and 95% of fallow deer samples) of the samples, with the best discriminator being the mean pellet length. Our results were different from those obtained by Spitzer et al. [11], where fallow deer and roe deer strongly overlapped for all measurements except pellet group size. Nevertheless, in that study, samples of the same species were taken from geographically distant populations, and differences in body size (and thus feces measurements) could have affected the results. Therefore, geographical variation in the body size of the two species needs to be taken into account when applying the method in practical monitory.

**Figure 4:**
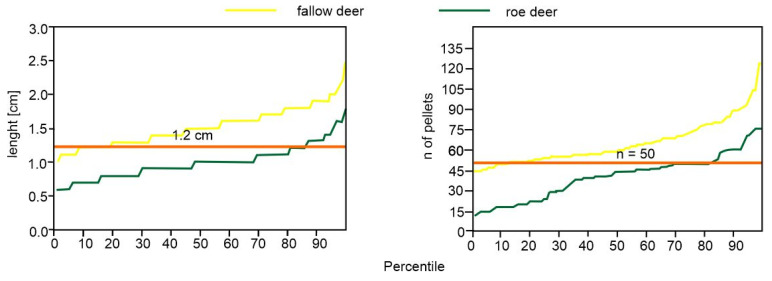
Percentile plots for the length of a pellet and the number of pellets in a group for the feces of roe deer and fallow. The line shows the value proposed to best differentiate between pellets of the two species. In the case of the number of pellets in a group the threshold ranges between 50 (indicated by percentile analysis) and 52 (indicated by the regression model).

In general, in our case, i.e., roe and fallow deer populations from central Europe, it was possible to indicate a metrical value for distinguishing pellets of both species, i.e., the pellet length. As shown by the percentile analysis, the most accurate threshold was 1.2 cm, i.e., only 12% of roe deer pellets were over this length value, while 15% of fallow deer pellets were below 1.2 cm (Figure 4). A similar result (1.2 cm separating the species) was obtained in the logistic regression model (Model 6, Figure 3). 

As Shown by Spitzer et al. [11], the commonly used threshold of 45 pellets in a group separated the means for roe and fallow deer. However, 30% of the fallow deer samples fell below that threshold, and 34% of the roe deer samples fell above. As we demonstrated on a percentile plot (Figure 4), a value of 50 pellets better differentiated between the two species. Nevertheless, the regression model indicated a slightly higher threshold of 52 pellets. Therefore, uncertainty between 50 and 52 occurs, which lowers the accuracy of species identification. Still, the pellets number below 50 (i.e., for roe deer) and higher than 52 (i.e., for fallow deer) appear to be accurate in the identification of a given species. Combining these two measurements (i.e., pellet length and the number of pellets in a group) might be an objective method to distinguish between pellet groups of the two species.

We propose that, in practice, field workers could evaluate the number of pellets in a group and conduct length measurements of at least five randomly selected pellets in a group. Collecting feces in order to perform the measurements in a laboratory is not recommended because transport and storage may distort the pellets. During eight hours of fieldwork, a team of two people can evaluate 150–250 pellet groups, depending on the dung density in the field [27]. 

The proposed procedure significantly increases the time and effort involved in the method of pellet group counting but provides more reliable and repeatable results than subjective (expertise) assessment. We performed the measurements in spring and we recommend to do the same in other studies. Indeed, as shown earlier, the overall identification success of pellets of ungulates was shown to decline from spring (90%) to summer and autumn (approx. 70%) before increasing again in winter (83%) [11]. Moreover, the season produces strong changes in red deer and fallow deer dung morphology [45].

## 4. Conclusions

To conclude, on the basis of morphometric measurement, it is possible to distinguish roe and fallow deer feces. The method should be applied in early spring, and the two values that best differentiated between pellet samples were pellet length and the number of pellets in a group. As a variation in the body size of the two species may affect measurements, the proposed thresholds should be treated with caution (and preferably confirmed in the field) when applied to geographically distant populations.

## Figures and Tables

**Table 1:** Relations of feces measurements (N of pellets = 500) of roe deer and fallow deer based on three generalized linear mixed models, with pellet group ID (N = 100) as a random effect (for all models fallow deer was a reference category in the SPECIES variable).

Model No. (Dependent Variable)	Source	B	SE	*p*	Lower CI	Upper CI
1 (LENGTH)						
	Intercept	−0.72	0.11	0.000	−0.92	−0.51
	SPECIES	1.03	0.05	0.000	0.94	1.13
	WIDTH	0.92	0.04	0.000	0.84	1.00
	WIDTH*SPECIES	−0.81	0.06	0.000	−0.92	−0,69
2 (WIDTH)						
	Intercept	−0.94	0.12	0.000	−1.17	−0.71
	SPECIES	0.47	0.07	0.000	0.34	0.60
	LENGTH	0.63	0.03	0.000	0.57	0.69
	LENGTH*SPECIES	−0.45	0.05	0.000	−0.54	−0.35
3 (nPELLETS)						
	Intercept	3.63	0.26	0.000	3.12	4.14
	SPECIES	0.54	0.02	0.000	0.5	0.57

**Table 2:** The effects of feces measurements on the separation of species (i.e., roe deer and fallow deer) in logistic regression N = 997 for models 4 and 5, N = 201 for models 6 and 7 (The RAW_A_ explanatory variables were the raw data of LENGTH, nPELLETS and WIDTH. The RAW_B_ explanatory variables were the raw data of LENGTH and nPELLETS. The MEAN_A_ explanatory variables were mLENGTH as the mean calculated for five pellets selected from a group and nPELLETS. The MEAN_B_ explanatory variable was only the mLENGTH mean calculated for five pellets selected from a group. The MEAN_C_ explanatory variable was only nPELLETS. D—the percentage of deviation explained by the model. OR—the odds ratio).

Model No. (Name)	Source	B	SE	*p*	OR
4 (RAW_A_)					
D = 71.32%	Intercept	−13.26	1.06	0.000	0.00
	nPELLETS	0.08	0.01	0.000	1.08
	LENGTH	10.61	0.82	0.000	40,382.14
	WIDTH	−5.07	0.98	0.000	0.01
5 (RAW_B_)					
D = 69.86%	Intercept	−15.34	1.01	0.000	0.00
	nPELLETS	0.85	0.01	0.000	1.01
	LENGTH	8.60	0.64	0.000	5431.43
6 (MEAN_A_)					
D = 78.07%	Intercept	−21.61	3.70	0.000	0.00
	nPELLETS	0.11	0.03	0.000	0.12
	mLENGTH	12.59	2.36	0.000	293,213.11
7 (MEAN_B_)					
D = 77.93%	Intercept	−15.48	2.34	0.000	0.00
	mLENGTH	12.54	1.88	0.000	278,144.85
8 (MEAN_C_)					
D = 56.87%	Intercept	−6.95	1.10	0.000	0.00
	nPELLETS	0.13	0.02	0.000	1.14

## Data Availability

The data presented in this study are available on request from the corresponding author.

## Supplementary Materials

The following supporting information can be downloaded at: https://www.mdpi.com/article/10.3390/ani12030290/s1, Table S1: Results of the Shapiro–Wilk W test for pellets of roe deer and fallow deer. The number of pellets was assessed for 100 pellet groups assigned to each species. Pellet length and width were estimated for five pellets from each of 100 pellet groups of the species; Figure S1: Feces of roe deer; Figure S2: Feces of fallow deer. Table S2: Ranking of generalized linear mixed models (including null model) explaining relations of feces measurements of roe deer and fallow deer with pellet group ID as a random effect to account for repeated sampling within groups.
