# Supplementary material for: Alien or Native? How to Distinguish Feces of Fallow and Roe Deer Using Central Poland as a Case Study
Source: Animals (Basel). 2022 Jan 25;12(3):290. doi: 10.3390/ani12030290 (PMC8833327; doi:10.3390/ani12030290)
Supplement: Supplementary file 1 [file animals-12-00290-s001.zip › Supplementary materials 1.pdf]

Supplementary

1. To check if data was normally distributed we perfomed Shapiro-Wilk W test for the three measurements (i.e. pellet length, width and the number of pellets in a group) for the two species (i.e. roe deer and fallow deer) separately (Table Suppl. 1). In all cases data did not follow normal distribution.

Table S1. Result of the Shapiro-Wilk W test for pellets of roe deer and fallow deer. The number of pellets was assessed for 100 pellet groups assigned to each species. Pellet length and width were estimated for five pellets from each of 100 pellet groups of the species.

Measurement	roe deer		fallow deer	
	W	p-value	W	p-value
length	0.9473	2.79E-12	0.9628	5.79E-10
width	0.9114	1.99E-16	0.9305	1.60E-14
n of pellets/group	0.9538	0.00131	0.8985	1.20E-06

2. Photographs of feces of roe deer and fallow deer. Feces of roe deer are shorter, narrower and less elongated than that of fallow deer.

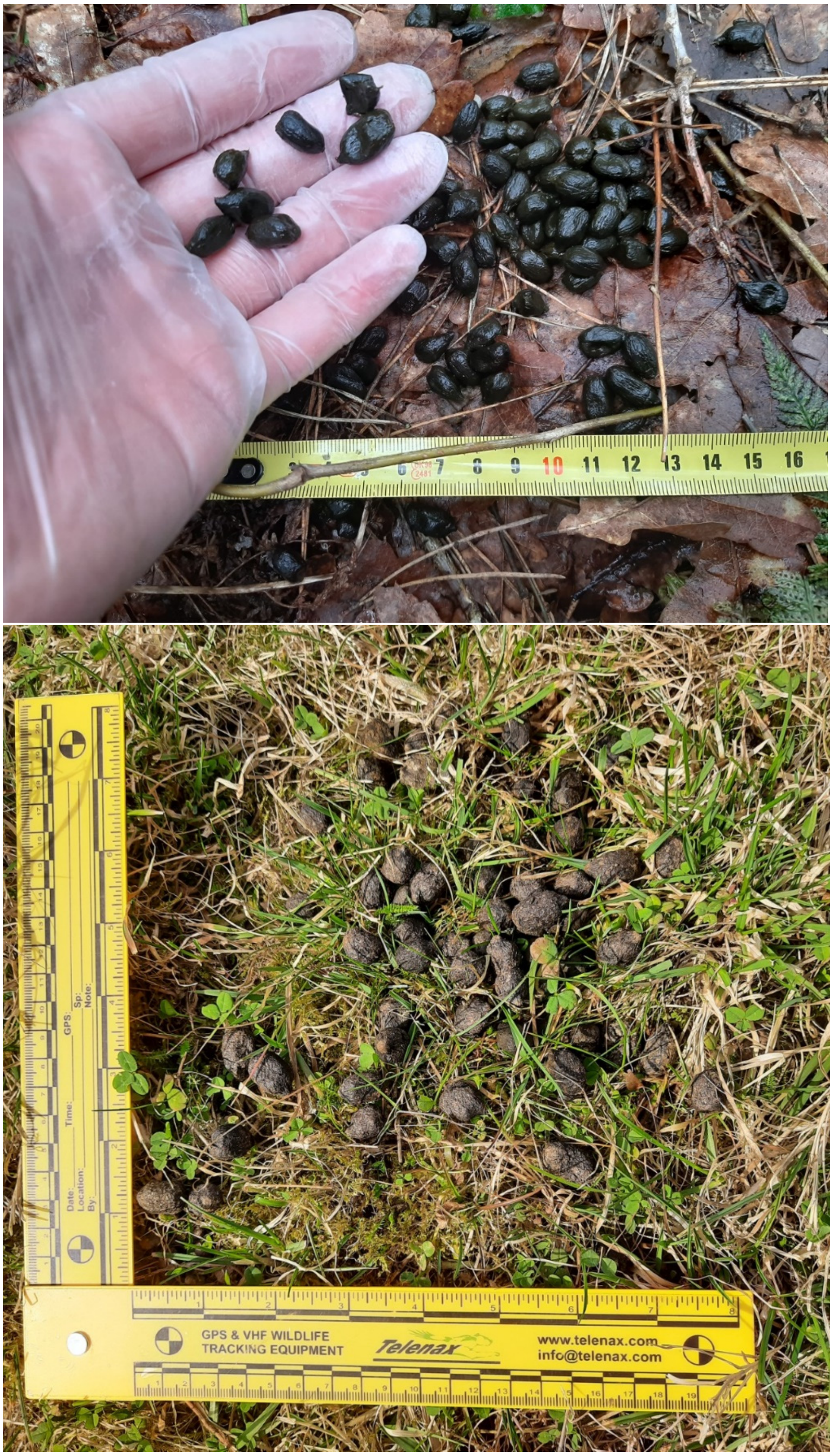

Figure S1. Feces of roe deer

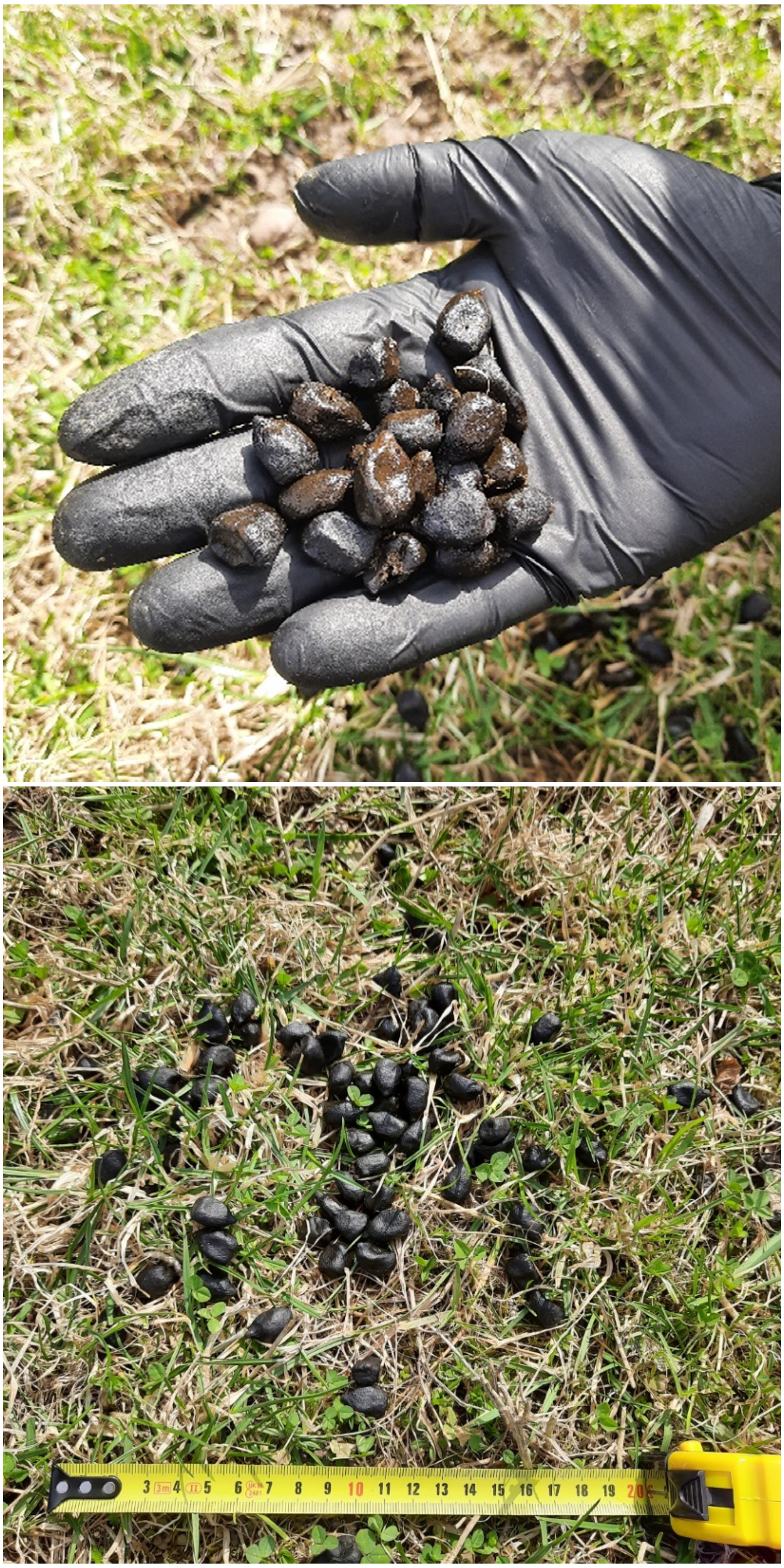

Figure S2. Feces of fallow deer.

Table S2. Ranking of generalized linear mixed models (including null model) explaining relations of feces measurements of roe deer and fallow deer with pellet group ID as random effect to account for repeated sampling within groups, Rank – rank of the models based on *AICc* values); variables: SPECIES: roe deer and fallow deer, LENGTH: feces length, WIDTH: feces width, nPELLETS: number of pellets in a group, LENGTH*SPECIES: interaction of length and species, WIDTH*SPECIES: interaction of width and species; best model in bold.

Model No (dependent variable)	Variables	*ΔAICc*	Rank
1 (LENGTH)	**SPECIES + WIDTH + WIDTH*SPECIES**	**0.00**	**1**
	SPECIES + WIDTH + nPELLETS + WIDTH*SPECIES	13.73	2
	SPECIES + WIDTH	148.52	3
	SPECIES + WIDTH + nPELLETS	161.68	4
	SPECIES	402.39	5
	SPECIES + nPELLETS	416.10	6
	WIDTH + nPELLETS	779.75	7
	nPELLETS	1061.77	8
	WIDTH	1129.50	9
	Null model	1379.95	10
2 (WIDTH)	**SPECIES + LENGTH + LENGTH *SPECIES**	**0.00**	**1**
	SPECIES + LENGTH + nPELLETS + LENGTH *SPECIES	6.70	2
	SPECIES + LENGTH	71.31	3
	SPECIES + LENGTH + nPELLETS	79.00	4
	LENGTH + nPELLETS	92.54	5
	LENGTH	102.42	6
	SPECIES	315.92	7

	SPECIES + nPELLETS	327.24	8
	nPELLETS	410.35	9
	Null model	439.24	10
3 (nPELLETS)	SPECIES	**0.00**	**1**
	SPECIES + WIDTH	3.42	2
	SPECIES + LENGTH	3.77	3
	SPECIES + LENGTH + WIDTH	4.30	4
	LENGTH + WIDTH	158.45	5
	LENGTH	169.84	6
	WIDTH	493.68	7
	Null model	530.39	8
